# Efficient Mating-Type Switching in *Candida glabrata* Induces Cell Death

**DOI:** 10.1371/journal.pone.0140990

**Published:** 2015-10-22

**Authors:** Stéphanie Boisnard, Youfang Zhou Li, Sylvie Arnaise, Gregory Sequeira, Xavier Raffoux, Adela Enache-Angoulvant, Monique Bolotin-Fukuhara, Cécile Fairhead

**Affiliations:** 1 Institut de Génétique et Microbiologie, Université Paris-Sud, UMR8621 CNRS, F-91405, Orsay, CEDEX, France; 2 Génétique Quantitative et Évolution–Le Moulon, INRA–Université Paris-Sud–CNRS–AgroParisTech, Batiment 400, UFR des Sciences, F 91405, Orsay, CEDEX, France; 3 Hôpital de Bicêtre, Le Kremlin Bicêtre, APHP, France; Louisiana State University, UNITED STATES

## Abstract

*Candida glabrata* is an apparently asexual haploid yeast that is phylogenetically closer to *Saccharomyces cerevisiae* than to *Candida albicans*. Its genome contains three *MAT*-like cassettes, *MAT*, which encodes either *MATa* or *MATalpha* information in different strains, and the additional loci, *HML* and *HMR*. The genome also contains an *HO* gene homolog, but this yeast has never been shown to switch mating-types spontaneously, as *S*. *cerevisiae* does. We have recently sequenced the genomes of the five species that, together with *C*. *glabrata*, make up the *Nakaseomyces* clade. All contain *MAT*-like cassettes and an *HO* gene homolog. In this work, we express the *HO* gene of all *Nakaseomyces* and of *S*. *cerevisiae* in *C*. *glabrata*. All can induce mating-type switching, but, despite the larger phylogenetic distance, the most efficient endonuclease is the one from *S*. *cerevisiae*. Efficient mating-type switching in *C*. *glabrata* is accompanied by a high cell mortality, and sometimes results in conversion of the additional cassette *HML*. Mortality probably results from the cutting of the *HO* recognition sites that are present, in *HML* and possibly *HMR*, contrary to what happens naturally in *S*. *cerevisiae*. This has implications in the life-cycle of *C*. *glabrata*, as we show that efficient *MAT* switching is lethal for most cells, induces chromosomal rearrangements in survivors, and that the endogenous *HO* is probably rarely active indeed.

## Introduction

Sexual reproduction in fungi takes diverse forms [1], some species preferring out-breeding and others inbreeding, either because spores resulting from meiosis conjugate inside the ascus, before germination, or because they mate with daughter cells, through homothallism [2]. Homothallism is formally defined by the fact that a single spore isolated after meiosis is able to undergo a full sexual cycle, without needing to meet a spore from the opposite mating-type. This is in opposition to heterothallism, where conjugation of cells from strains of opposite mating-types is required. Different underlying mechanisms for homothallism exist [1,3,4], especially in higher fungi, but the best studied such mechanism in yeasts, is the mating-type interconversion mechanism from the model yeast *Saccharomyces cerevisiae* [5]. It is interesting to note that the haploid *Schizosaccharomyces pombe*, the other model yeast favored by scientists, also switches mating-types very efficiently. The two species use different initiation events, but undergo equivalent gene conversion events, in an example of convergent evolution [6,7]. Both mechanisms involve potentially dangerous chromosomal breaks, in pathways which are, by necessity, finely regulated. It is notable that the *HO* gene, which encodes the endonuclease central to the process in *S*. *cerevisiae*, has, indeed, a large regulatory region upstream [8]. Several levels of regulation control the expression of *HO*: it is only expressed in haploid cells, only in late G1 and only in mother cells [8,9].

In *S*. *cerevisiae*, cellular mating-type is determined by the *MAT* locus, which encodes transcription factors responsible for sexual identity [10–13]. Three types of sexual identity exist; mating-competent haploid *MATa* and *MATalpha* cells, according to the sequence present at the *MAT* locus, and meiosis-competent diploid cells which contain active copies of both types. Cells also contain transcriptionally silent copies of both mating-types [14,15] which are bordered by identical sequence segments that drive homologous recombination [16–18]. Mating-type interconversion in wild-type homothallic haploid cells relies on two components: the *MAT*-like loci and the Ho endonuclease (for review [5]). After the daughter cell has budded, the mother cell's active *MAT* locus is cut by the Ho endonuclease at its recognition site, at the junction between the Y and Z1 segments of the locus [19–21]. This double strand break is repaired by homologous recombination using the *HMLalpha* or the ***HMRa*** cassette as a template for repair (for review [5,22]), resulting in a switch of mating-types. Switching is highly efficient, with 90% cells switching to the opposite sexual type [3,23,24], through the control by the sexual identity of the cell, *MATalpha* cells repressing the Recombination Enhancer present between the *MAT* and the *HML* loci, leading to repair with *HMR* [5,25,26]. In this process, the *MAT* locus is the only one to be cut, *HMLalpha* and ***HMRa*** being protected from the Ho cleavage by silencing processes involving the Sir proteins [27–29].

Asexual species abound in the fungal kingdom, and it has been noted that fungal pathogens of humans usually display an apparent lack of sexuality. The reasons for this include the absence of cells of one mating-type in an infectious population of a heterothallic species, such as in the case of *Aspergillus fumigatus* [30], or modification of mating pathways to yield atypical, rare, conjugation events between diploids in addition to conjugation events between even rarer haploids, such as in the case of *C*. *albicans* [31–33].

*C*. *glabrata*, the 2^nd^ cause of invasive candidiasis after *C*. *albicans* [34], has never been shown to mate, but haploid cells of both mating-types are regularly isolated [35], and the genome contains both the triplicated *MAT*-like loci and an *HO* gene homolog [36,37]. We have previously shown that *C*. *glabrata MATalpha* cells express the *MATa* specific gene, *MATa1*, through transcriptional “leakage” of ***HMRa***, and that both *C*. *glabrata MATa* and *MATalpha* cells are insensitive to their specific mating pheromones, while *S*. *cerevisiae*'s cells are sensitive to the same pheromones [38].

*C*. *glabrata* belongs to the *Nakaseomyces* genus, which includes five other species, two described recently as pathogens, *Candida* (*Nakaseomyces*) *nivariensis* [39] and *Candida* (*Nakaseomyces*) *bracarensis* [40]; and three species isolated in the environment: *Kluyveromyces* (*Nakaseomyces*) *delphensis*, *Kluyveromyces* (*Nakaseomyces*) *bacillisporus*, and *Candida* (*Nakaseomyces*) *castellii* [41]. This group of yeasts is closer to *Saccharomyces cerevisiae* than to *Candida albicans*, and therefore follows the universal nuclear genetic code. All *Nakaseomyces* species are haploid, except for *N*. *bacillisporus*, which is diploid [41,42]. We have recently sequenced the genomes of these five species, and compared them to *C*. *glabrata* [42]. Contrary to *S*. *cerevisiae*, where the three loci are on chromosome III, in *C*. *glabrata*, *HMLalpha* and *MAT* are on chromosome II, and ***HMRa*** is on chromosome V [43]. This configuration is common to four species in the *Nakaseomyces*, while one, *N*. *delphensis*, has the three loci on the same chromosome like *S*. *cerevisiae*. As for the final species, *N*. *bacillisporus*, the sequence in its present state does not contain the *HMR* locus.

*C*. *glabrata* has an *HO* gene homolog, where known domains for nuclear localization, site recognition and endonuclease activity are conserved [44]. This is also true in all *Nakaseomyces* [42]. Mating-type switching has been suggested by PCR experiments in *C*. *glabrata* [45], but no living cell where spontaneous switching has occurred and been followed experimentally has been isolated, such as is possible in *S*. *cerevisiae*, where *HO* haploid cells switch every generation, and pedigrees of cells can be performed to follow this. Recently, induction of *HO* expression inducing mating-type switching in *C*. *glabrata* has been reported, using a constitutive promoter and the endogenous gene, resulting in switching of a *MATalpha* cell to a ***MATa*** cell [46]. Populations of *C*. *glabrata* contain strains of both mating-types with some collections exhibiting bias [35,47], and it has been reported that some strains exhibit “abnormal” cassette configurations, *i*.*e*, differing from the canonical *HMLalpha* and ***HMRa*** configuration [36]. This is also known in *S*. *cerevisiae* [48,49].

It has been proposed that triplicated *MAT*-like cassettes originated before the *HO* gene, and that these were used to allow rare mating-type switching events, and that the *HO* gene, a “selfish” self-transposing gene, was then “domesticated” [50–52]. Indeed, *K*. *lactis* switches mating-types inefficiently through mechanisms independent of Ho but dependent on triplicated cassettes [53,54]. The *HO* gene is part of the family of Homing Endonuclease Genes (HEGs), selfish genetic elements that can propagate through populations. *HO* is closely related to *VDEI*, the intein in *S*. *cerevisiae's* genome [55]. Many HEGs propagate as self-splicing introns in organelle genomes; the intron encodes an endonuclease that can cut its site in a genome without the intron, upon mating of an intron-containing and an intron-less strain. The cut is then repaired by homologous recombination (HR), using the intron-containing gene as template, resulting in intron propagation [56]. Ho is an intein whose sole known activity in the cell is to cut its recognition site at *MAT* and that is encoded by a free-standing gene, dissociated from its own recognition site on chromosome III. Endonucleases of the Ho family have rather low activity, recognize large sites but tolerate degenerate bases within the site, as shown by mutagenesis of the Ho and I-*Sce* I recognition sites [20,21,57].

We have previously published the composition of *MAT*-like cassettes and comparison of the *HO* genes in the *Nakaseomyces* [42]. We now report on heterologous expression of *HO* from different *Nakaseomyces* species and from *S*. *cerevisiae* in *C*. *glabrata*, and the resulting switching events in both directions, with abnormal structures resulting from gene conversion events. We observe that a high frequency of switching is associated with cell mortality.

## Materials and Methods

### Strains, cultures and transformation

Strains used for *in vivo* experiments and for amplification of *HO* genes are listed in Table 1. Yeast strains are grown in broth or on plates at 28°C, in YDP (non-selective, 1% Yeast Extract, 1% Peptone, 2% glucose), Synthetic Complete medium lacking uracil (SC-Ura, 0.67% Yeast Nitrogen Base without amino acids, 2% glucose, supplemented with all amino acids and adenine) for *HO* induction experiments. For selection of transformants and maintenance in repressive conditions, strains are grown in SC-Ura added with 2mM each of methionine and cysteine. Stability of plasmids in *C*. *glabrata* and *S*. *cerevisiae* was confirmed by comparing the number of colonies on SC-Ura and SC+Ura. Transformation of *S*. *cerevisiae* and *C*. *glabrata* was done according to the lithium acetate transformation protocol from Gietz *et al* [58].

**Table 1:** Strains of *Nakaseomyces* and *S*. *cerevisiae* for amplification of *HO* genes and yeast transformation.

Species	Strain	Genotype	Reference
*S*. *cerevisiae*	J5	*HML*alpha, *MAT*alpha, *HMR*alpha, *HO*,*leu2*, *his4*, *thr4*, *lys2*	Kindly donated to us by Amar Klar
*S*. *cerevisiae*	W303-1B	*ho*, ***MATa***	ATCC 200060
*S*. *cerevisiae*	FY69	*ho*, ***MATa***, *leu2Δ1*	[59]
*S*. *cerevisiae*	FYC2-7B	*ho*, *MATalpha*, *leu2Δ1*	[60]
*S*. *cerevisiae*	FY1679-18D	*ho*, *ura3-52*, *his3Δ200*	[60]
*S*. *cerevisiae*	FY73	*ho*, *ura3-52*, *his3Δ200*	[59]
*N*. *delphensis*	CBS2170	Type strain, 1n, actively switching *	[42]
*N*. *bacillisporus*	CBS7720	Type strain, 2n	[42]
*C*. *castellii*	CBS4332	Type strain, 1n, probably switching *	[42]
*C*. *bracarensis*	CBS10154	Type strain, 1n, MATalpha	[42]
*C*. *nivariensis*	CBS9983	Type strain, 1n, MATalpha	[42]
*C*. *glabrata*	CBS138	Type strain, 1n, MATalpha	[42]
*C*. *glabrata*	BG2	1n, **MATa**	[61]
*C*. *glabrata*	BG87	**MATa**, NeoR::ura3, his3Δ	[62]
*C*. *glabrata*	HM100	*MATalpha*, *URA3Δ*::*KANMX*	[38]

* our unpublished results

It must be noted that the *S*. *cerevisiae* strain used for *HO* induction has a mutant endogenous *HO* gene; while the *C*. *glabrata* strains still possess their endogenous *HO* gene.

### Cloning of *HO* genes into expression plasmids for *C*. *glabrata*

The different *HO* genes were amplified by PCR from genomic DNA on type strains for the *Nakaseomyces* and on an *HO* ^*+*^ strain in the case of *S*. *cerevisiae* (Tables 1 and 2). *Spe*I/ *Sal*I sites were added to primers used for *HO* gene amplification. Appropriate PCR fragments were cloned at the *Spe*I/*Sal*I sites into the pYR32 plasmid. The pYR32 plasmid, kindly provided by B. Cormack [63], is a replicative plasmid for *C*. *glabrata*, and was also used for transformation of *S*. *cerevisiae*. Absence of mutation in all the *HO* genes (amplified and cloned) was verified by sequencing.

**Table 2:** Primers used for PCR amplification of *HO* gene and PCR probe amplification.

Primers	Species name	Primer sequence (5'-3')
RX1-KLDE-F	*N*. *delphensis*	GCATACTAGTATGTTTGACATTAACACAAC
RX2-KLDE-R	*N*. *delphensis*	CGATGTCGACCTAATTTATCATAGCACGCC
RX3-CANI-F	*C*. *nivariensis*	GCATACTAGTATGTTTGAGATAAATACAAC
RX4-CANI-R	*C*. *nivariensis*	CGATGTCGACCTAGTTCAGCATAACAC
RX5-CACA-F	*C*. *castellii*	GCATACTAGTATGTTAGAAGAGAAAACTCA
RX6-CACA-R	*C*. *castellii*	CGATGTCGACTTAACATGCTTCTAATGCAA
RX7-CABR-F	*C*. *bracarensis*	GCATACTAGTATGTTTGAAAAAAATACTAC
RX8-CABR-R	*C*. *bracarensis*	CGATGTCGACTTAATTAAGCATAGCGTG
RX9-KLBA-F	*N*. *bacillisporus*	GCATACTAGTATGTTAGAAGAAAATACCCA
RX10-KLBA-R	*N*. *bacillisporus*	CGATGTCGACCTATATATAGACAGATGAAT
RX11-CAGL-F	*C*. *glabrata*	GCATACTAGTATGTTCGAAAAGGGAACTTT
RX12-CAGL-R	*C*. *glabrata*	CGATGTCGACAATAGCGGATGTACTCTATT
RX13-SACE-F	*S*. *cerevisiae*	GCATACTAGTATGCTTTCTGAAAACACGAC
RX14-SACE-R	*S*. *cerevisiae*	CGATGTCGACTACACATTTTAGCAGATGCG
A1 probe F	*C*. *glabrata*	CCAATACAAGATCTACGCA
A1 probe R	*C*. *glabrata*	GATCTCTTGCGCCTATTTG
ALPHA1 probe F	*C*. *glabrata*	ACTGAAACACTGACTATGAAG
ALPHA1 probe R	*C*. *glabrata*	CTGAGAGAATGACGGAGAG

### PCR, Southern Blot and Sequencing

To test mating-type switching, we performed PCR on colonies before and after induction, using specific primers, which can discriminate the mating-type at the *MAT* locus, by amplifying the upstream part of the locus (Tables 3 and 4). In some cases, we also typed *HML* and *HMR* (Tables 3 and 4, and see results). Cells from a fresh colony were incubated in 5 μL of NaOH 20 mM 5 min at 95°C in the PCR tubes before placing at 4°C, adding the buffer, primers, dNTPs and Taq polymerase and proceeding with the PCR amplification.

**Table 3:** Location and sequence of primers used for determining mating-type information at *MAT*, *HML*, and *HMR*.

Name	Primers	Localisation
GS01	TACCAAGAAGCAAGAGCCCA	Upstream of *MAT*
GS02	TCTTGCGTAGTCGAGACCTC	Downstream of *MAT*
GS06	GACAGGAACATCTAAGCGAT	Upstream of *HMR*
GS07	GTGATGATTACTGGGTGGA	Downstream of *HMR*
GS08	GCTGTAGTGGCGAAAATAAG	Upstream of *HML*
GS09	GATCACTTCGTAGTAGAAAAC	Downstream of *HML*
RX15	GCTGATCGAGGTGAATCCAT	Upstream of *MAT*
RX16	CTCTACCAGCAAAGGCCAAG	Inside Ya *(MAT*, *HML*, *HMR)*
RX17	TTCAACCGCCTAAAAATTGC	Inside Yalpha *(MAT*, *HML*, *HMR)*

**Table 4:** Combination of primers used and size of expected products.

Primer pairs	Specific locus	Amplification length expected
RX15/RX16	Upstream ***MATa*** fragment	778bp
RX15/RX17	Upstream *MATalpha* fragment	1161bp
GS08/RX16	Upstream *HMLa* fragment	1042bp
GS08/RX17	Upstream *HMLalpha* f*ragment*	1425bp
GS06/RX16	Upstream ***HMRa*** fragment	1060bp
GS06/RX17	Upstream *HMRalpha* fragment	1443bp
GS01/GS02	Whole *MAT* locus	***MATa*** 2315bp, *MATalpha* 2500bp
GS08/GS09	Whole *HML* locus	*HMLa* 2297bp, *HMLalpha* 2482bp
GS06/GS07	Whole *HMR* locus	***HMRa*** 2297bp, *HMRalpha* 2482bp

For Southern blots, genomic DNAs were prepared using the Qiagen genomic DNA kit, according to manufacturer's instructions. 5 μg DNA was subjected to enzymatic digestions by *Eco*RV or *Hind*III/*Pst*I. Gels (0.8% agarose in 0.5x TBE) were transferred on positively charged Nylon membranes from Roche, using an Appligene vacuum blotter. Membranes underwent pre-hybridization for 4 hrs and overnight hybridization at 65°C in Church's buffer [64], with a DIG-labeled PCR probe, and were submitted to high stringency washes at 65°C in 0.1% SDS, 1X and then 0.1X SSC buffer. DIG labeling and detection was done using a Roche kit, according to manufacturer's instructions. Primers used for probe PCR amplifications are given in Table 2.

For the three cassettes *MAT*, *HML* and *HMR*, the whole locus was amplified using primers indicated on Tables 3 and 4, and purified PCR fragments were sequenced by Beckman Coulter Inc.

### Induction of *HO* expression and quantification of the efficiency of mating-type switching

**Fig 1:**
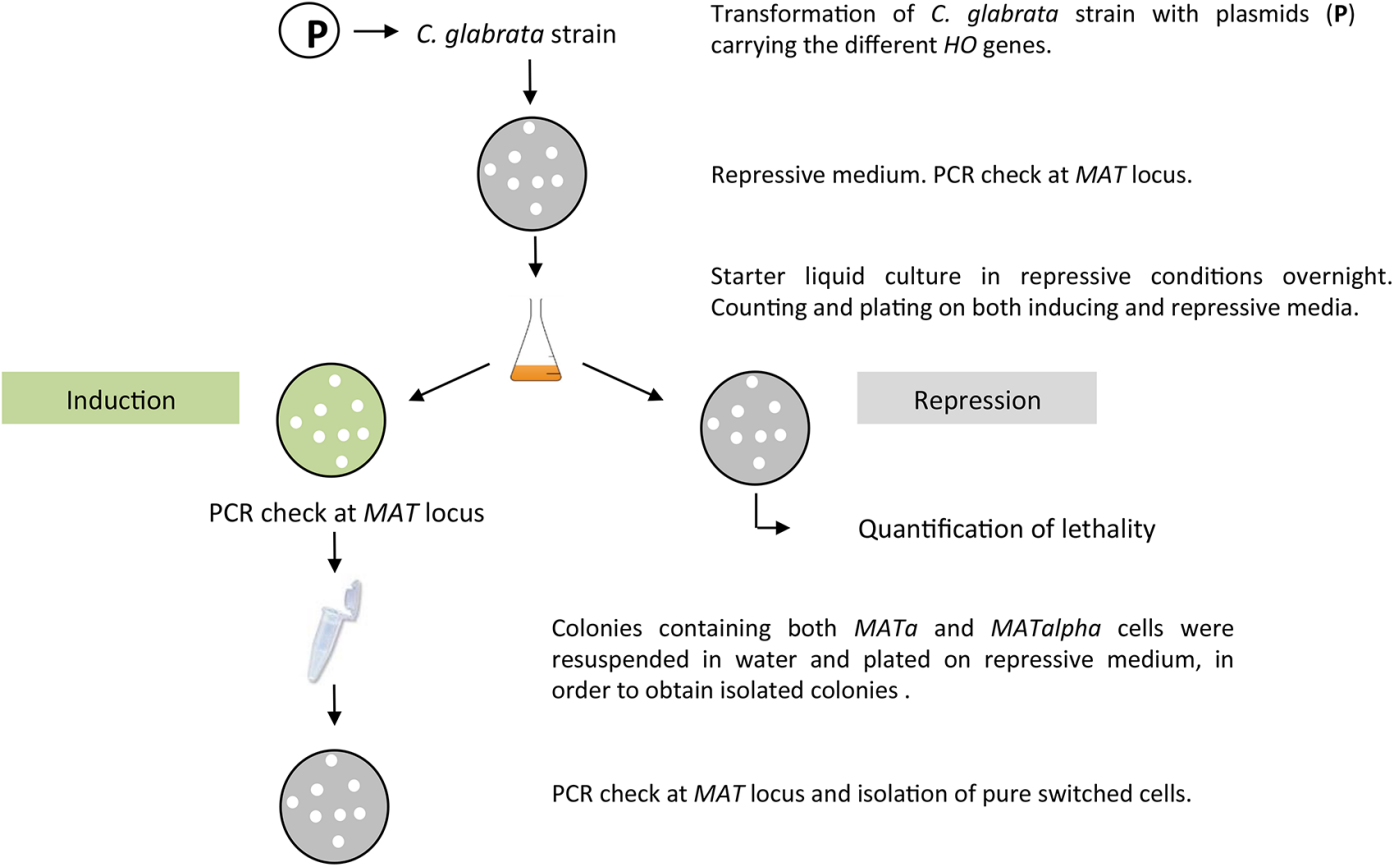
Experimental procedure for induction of the *HO* gene and isolation of switched cells.

Experimental procedure is shown on Fig 1. Transformants with the different *HO* plasmids were streaked onto repressive medium and directly tested by PCR (see above) to check that they had not switched before induction. Cells from a starter overnight culture in repressive conditions (SC-URA +2mM Met/Cys) were then counted, and around 200 cells were usually plated on inductive (SC-URA) and on repressive medium as control, and incubated at 28°C, until colonies appeared (one to two days). Determination of mating-type was done on individual colonies (a minimum of 14 colonies were tested per transformant plated, and at least 2 transformants were tested per experiment), by PCR amplification of both ***MATa*** and *MATalpha*.

At this step, we obtain "negative" colonies (those that had not switched and yielded a PCR product only with primers amplifying the original mating-type), and "mixed" colonies (colonies yielding a PCR product with both pairs of primers, in which some cells have switched, and others have maintained their original mating-type).

At least two "mixed" colonies were then restreaked on repressive medium, cells allowed to grown into individual colonies, and the PCR experiment performed again on at least 20 colonies per mixed colony. Colonies become "pure" as to their mating-type at this stage (*i*.*e*, no switching back and forth between mating-types occurs and all cells respond identically to the PCR determination, yielding a single positive response, either ***MATa*** or *MATalpha*). The percentage of "pure" switched colonies out of the number of colonies sub-cloned was then used as an estimate of the efficiency of mating-type switching (see results).

In the case of the expression of the *HO* gene from *S*. *cerevisiae*, we observed a high cellular mortality, so we decided to analyze this phenomenon more precisely: we plated dilutions from 10^6^ to 10^2^ cells on inductive medium and on repressive medium and compared the number of colonies growing. This was done on three and five independent transformants for strains BG87 and HM100 respectively.

For the induction of *HO* expression in BG87s, (BG87 switched from ***MATa*** to *MATalpha*) a colony which had lost the original plasmid was isolated by growing the strain on SC+URA, and transformed anew with the plasmid expressing the *HO* gene from *S*. *cerevisiae* (see Results).

## Results

### Mating-type switching in a *C*. *glabrata MATa* strain

*HO* is particularly well-conserved in the *Nakaseomyces* [42], and we cloned all *HO* genes from the *Nakaseomyces* and from *S*. *cerevisiae* into a *URA3* selectable plasmid that allows controlled expression of the cloned gene under the *MET3* promoter [63]. Expression is repressed in conditions of high methionine and cysteine concentrations, and induced in the absence of these two amino acids in the medium (see Materials and Methods).

**Fig 2:**
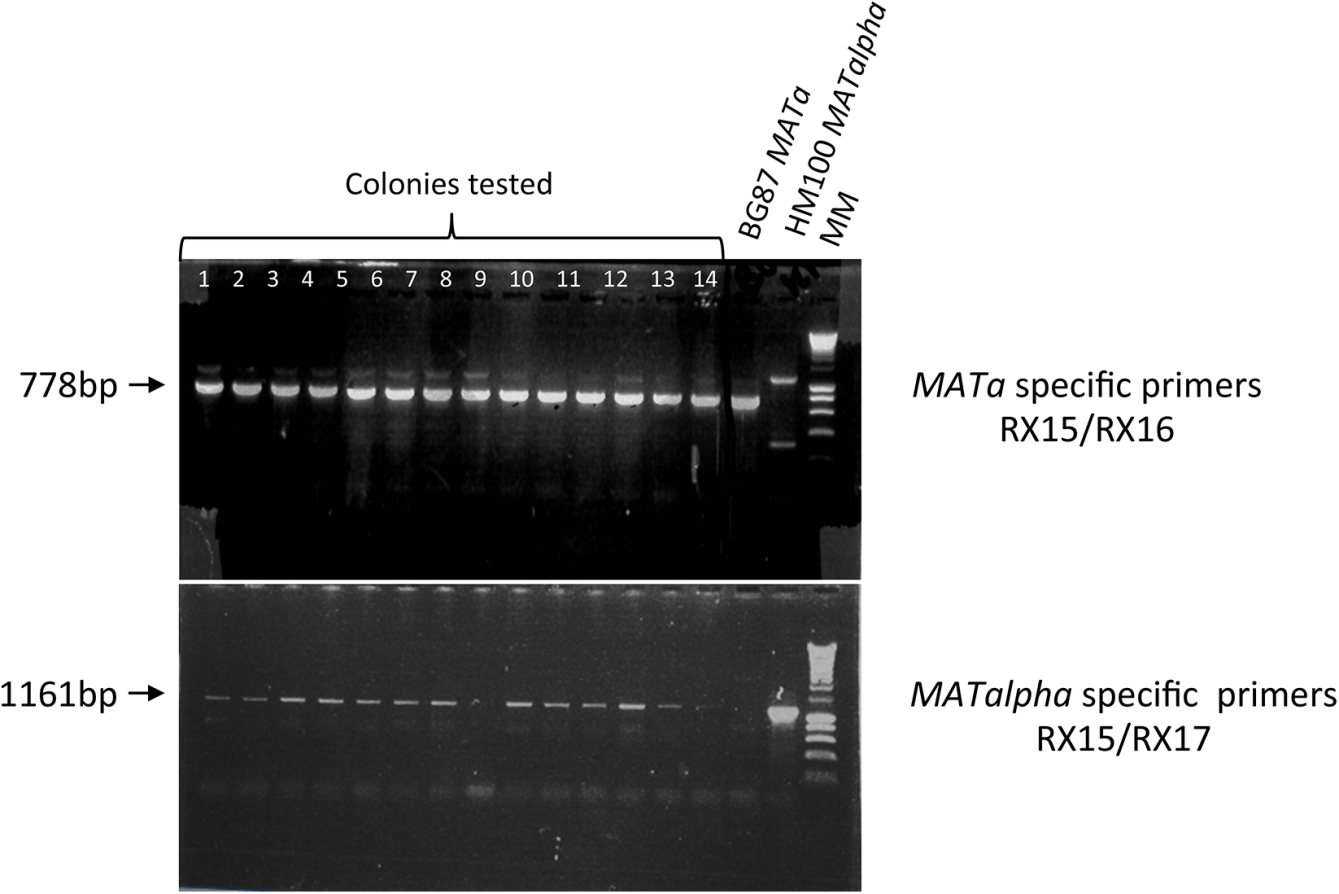
Expression of the *S*. *cerevisiae HO* gene in the *MATa C*. *glabrata* strain BG87. Top: amplifications obtained with ***MATa*** specific primers (RX15/RX16); Bottom: amplifications obtained with *MATalpha* specific primers (RX15/RX17). 13/14 colonies show both ***MATa*** and *MATalpha* specific amplification (except colony number 9). The BG87 ***MATa*** and HM100 *MATalpha* strains are used as controls.

We first tested the expression of all *HO* genes in *C*. *glabrata*, using the ***MATa*** strain BG87, isogenic to the BG2 strain, commonly used in laboratories [38,62] (Table 1). Colonies were tested by specific PCR in order to discriminate the mating-type (specific primers and corresponding size expected are given in Tables 3 and 4). Results obtained after induction are shown in Table 5 (experiment A and 1 to 7). All *HO* genes, from the *Nakaseomyces* and from *S*. *cerevisiae*, are able to induce mating-type switching, *i*.*e*, induction of *HO* expression yields “mixed colonies”, as evidenced by the obtainment of both ***MATa*** *-* and *MATalpha-*specific amplifications (see Materials and Methods). Fig 2 shows such PCR amplification in colonies after expression of the *HO* gene from *S*. *cerevisiae*; *i*.*e*, most of the colonies tested present both ***MATa*** *-* and *MATalpha*-specific amplifications.

**Table 5:** *MAT* switching in *C*. *glabrata* and in *S*. *cerevisiae*.

	Experiment	Strain	MAT	*HO* gene	Observation of switch	Pure switched cells	Molecular analysis of switch
*C*. *glabrata*	A	BG87	a	Without *HO* gene	-	Absent	ND
	1	BG87	a	*S*. *cerevisiae*	+	Frequent	Normal
	2	BG87	a	*C*. *glabrata*	+	Rare	Normal
	3	BG87	a	*C*. *bracarensis*	+	Rare	Normal
	4	BG87	a	*C*. *nivariensis*	+	Rare	ND
	5	BG87	a	*C*. *castellii*	+	Absent	ND
	6	BG87	a	*N*. *bacillisporus*	+	Rare	Normal
	7	BG87	a	*N*. *delphensis*	+	Rare	Normal
	8	HM100	alpha	*S*. *cerevisiae*	+	Frequent	Normal and Triple « a » strains
	1-b	BG87s	alpha	*S*. *cerevisiae*	+	Frequent	Normal and Triple « a » strains
*S*. *cerevisiae*	21	FY1679-18D	a	*S*. *cerevisiae*	+	Frequent	ND
	22	FY1679-18D	a	*C*. *glabrata*	-	Absent	ND

*HO* genes from different species (column 5) were expressed in strains of *C*. *glabrata* and *S*. *cerevisiae* (column 3). BG87s (experiment 1-b) corresponds to the BG87 strain switched to *MATalpha*. Switching events are detected by specific PCR (column 6); (+) indicates the presence of the opposite mating-type. After having isolated cells with pure genotypes (see materials and methods), estimation of the number of pure switched cells is referred in this table as absent, rare or frequent (column 7). Molecular analysis was performed by sequencing the *MAT*, *HML*, *HMR loci* (column 8). ND means Not Determined.

From these mixed ***MATa***/*MATalpha* colonies, we isolated pure switched cells (see Materials and Methods, and Fig 1). When *S*. *cerevisiae*’s *HO* gene is expressed, more than 80% of the isolated cells present the opposite sexual type (“frequent” in experiment 1, Table 5) whereas less than 10% of the isolated cells had switched when the different *Nakaseomyces HO* gene were expressed (“rare” in experiments 2, 3, 4, 6, and 7, Table 5). In the case of *N*. *castelli’s HO*, we could not isolate pure switched cells from a mixed colony exhibiting both ***MATa*** */MATalpha* amplification (“absent” in experiment 5, Table 5).

As a control (experiment A, Table 5), we performed the same experiment with the pYR32 plasmid which does not contain an *HO* gene and we never observed mixed ***MATa*** */MATalpha* colonies, but only non-switched parental colonies.

**Fig 3:**
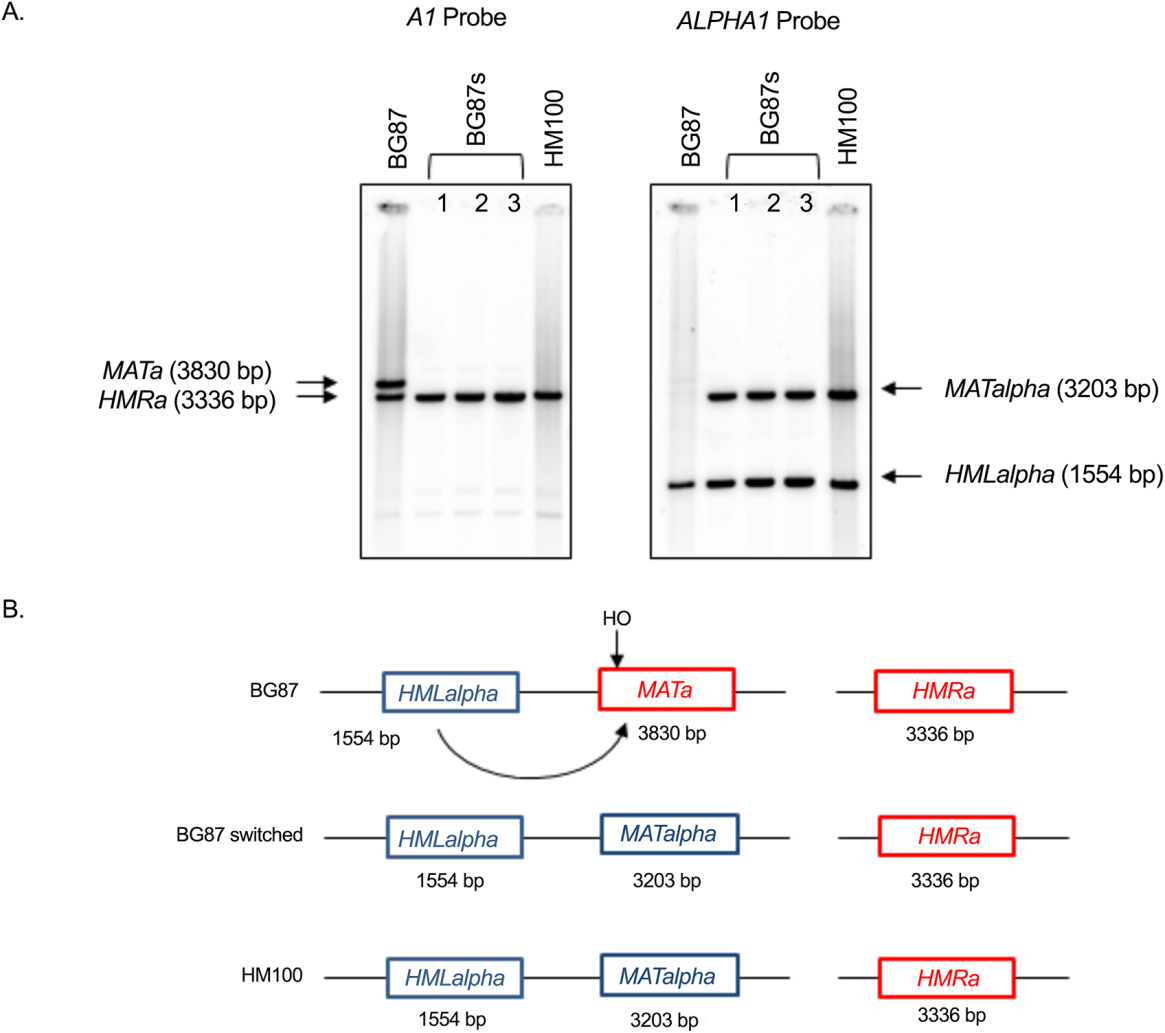
Southern autoradiogram analysis for strains BG87, switched BG87 (BG87s clones 1, 2, 3) and HM100 at the *MAT*, *HML*, and *HMR* loci. A) Southern autoradiogram analysis for strains BG87, switched BG87 (BG87s clones 1, 2, 3) and HM100 at the *MAT*, *HML*, and *HMR* loci. Genomic DNA was digested by *Hind*III/*Pst*I. BG87 (*HMLalpha*, *MATa*, ***HMRa***) and HM100 *(HMLalpha*, *MATalpha*, ***HMRa***) were used as controls. B) Diagram of the 3 cassettes for BG87 and HM100 and the switching event leading to strain BG87switched (BG87s).

**Fig 4:**
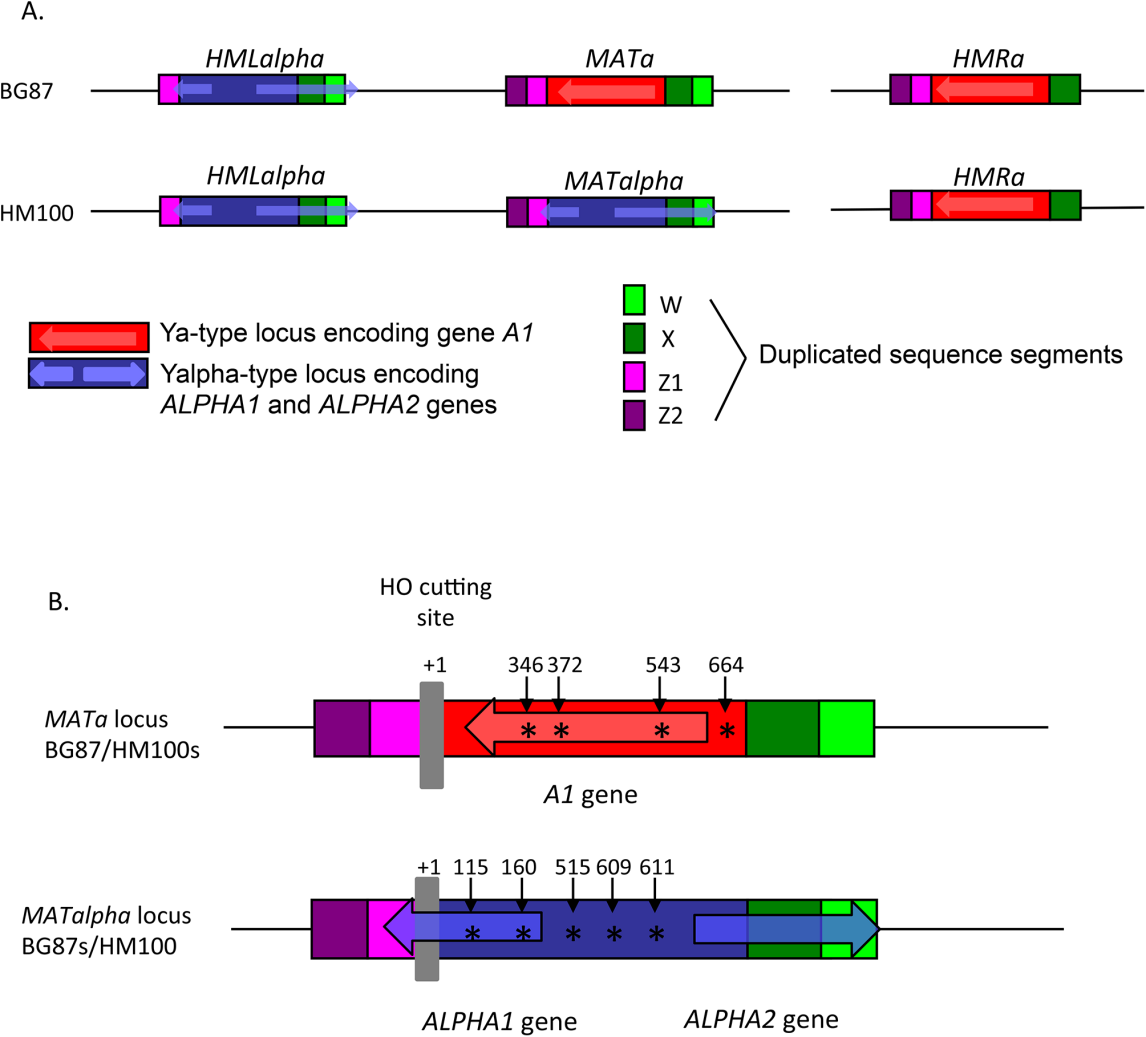
Structure of the *MAT* loci in *C*. *glabrata*. A. Structure of the *HML*, *MAT*, *HMR* loci in *C*. *glabrata*.B. Polymorphic sites at the *MAT* locus between BG87/HM100s and BG87s/HM100 strains. (+ 1) indicates the start of the *HO* recognition site. Polymorphic positions are indicated by asterisks.

Southern blot analysis (Fig 3) of pure switched strains shows that the structure of the cassettes is as expected after mating-type switching *i*.*e*, *HMLalpha*, *MATalpha*, ***HMRa***. The *MAT* locus from several pure switched clones (originating from expression of both *S*. *cerevisiae HO* and some *Nakaseomyces HO*s) was sequenced. This confirmed that the *C*. *glabrata MAT* locus undergoes switching from ***MATa*** to *MATalpha*. Sequence analyses show that the molecular structure of *MAT* locus is normal, suggesting a correct double strand break at the specific ***MATa*** *HO* cutting site and normal repair by homologous recombination with *HMLalpha*. Sequencing also confirmed that the switched clones obtained, truly originated from the BG87 strain, since several polymorphic sites at the *MAT* locus (discriminating the two *C*. *glabrata* background strains HM100 and BG87) were detected (Fig 4). *HMLalpha* and ***HMRa*** sequencing have shown that these loci were normal.

Since switching is observed at a high frequency only with *S*. *cerevisiae's HO*, and that all other give switching rates similar to *C*. *glabrata*'s *HO*, we focused on *S*. *cerevisiae HO* gene expression for further analyses.

### Mating-type switching in *C*. *glabrata MATalpha* strains

Contrary to *S*. *cerevisiae* where the three cassettes *HML*, *HMR* and *MAT* are on the same chromosome (ChIII), in *C*. *glabrata*, the *HMR* locus is located on a different chromosome from the *HML* and *MAT* loci. In *S*. *cerevisiae*, it is known that the relocation of *HMR* or *HML* lowers the efficiency of repair of the Ho cut [65,66]. We wanted to know whether switching occurred in the same way in both directions in *C*. *glabrata*. Our first experiments described above involved repair of the *MAT* locus with the *HML* locus on the same chromosome. In a *MATalpha* strain, such as HM100, switching involves repair with ***HMRa*** on a distinct chromosome. Switching from *MATalpha* to ***MATa*** has been performed previously, using the *HO* gene from *C*. *glabrata* expressed from a constitutive promoter [46], but was not examined in detail.

**Fig 5:**
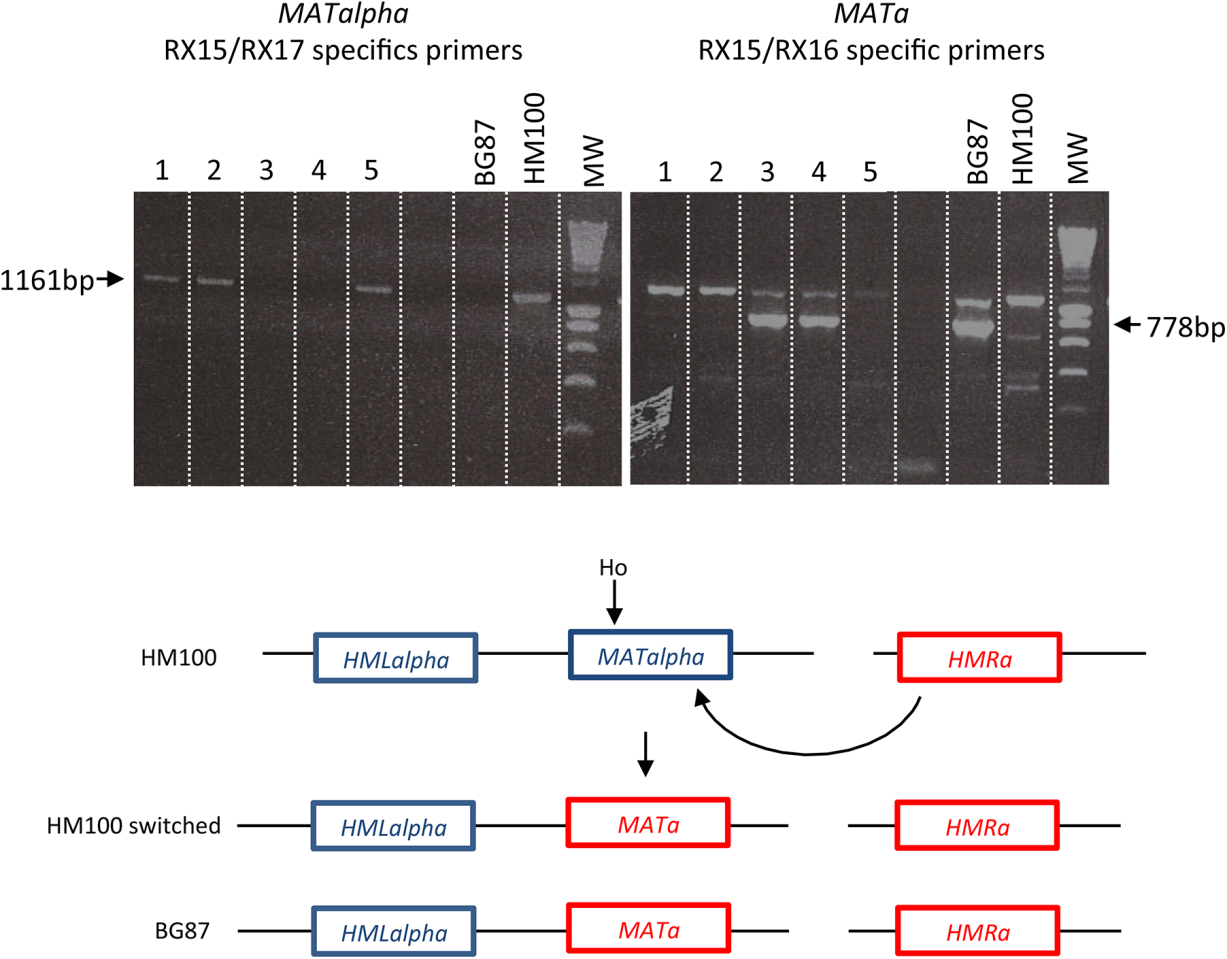
Expression of *S*. *cerevisiae HO* in the HM100 *MATalpha* strain of *C*. *glabrata*. Right panel: amplifications obtained with ***MATa*** specific primers (RX15/RX16), left panel: amplifications with *MATalpha* specific primers (RX15/RX17). Note: ***MATa*** primers amplify additional non-specific loci in both switched clones and in the HM100 control strain.

Therefore, expression of the *HO* gene from *S*. *cerevisiae* was induced in the *MATalpha* strain, HM100, isogenic to the sequenced strain, CBS138 (experiment 8, Table 5). Like previously, colonies grown on inductive medium were tested by specific PCR at the *MAT* locus (Fig 5). The expression of *S*. *cerevisiae’s HO* allows the obtainment of several pure switched subclones. Sequence analyses of these clones show that the *C*. *glabrata MAT* locus can switch from *MATalpha* to ***MATa***, and suggest again a correct double strand break at the *MATalpha* Ho cutting site and normal repair with ***HMRa*** as donor.

### Switching from *MATalpha* to *MATa* reveals unexpected cutting at *HML* in *C*. *glabrata*

**Fig 6:**
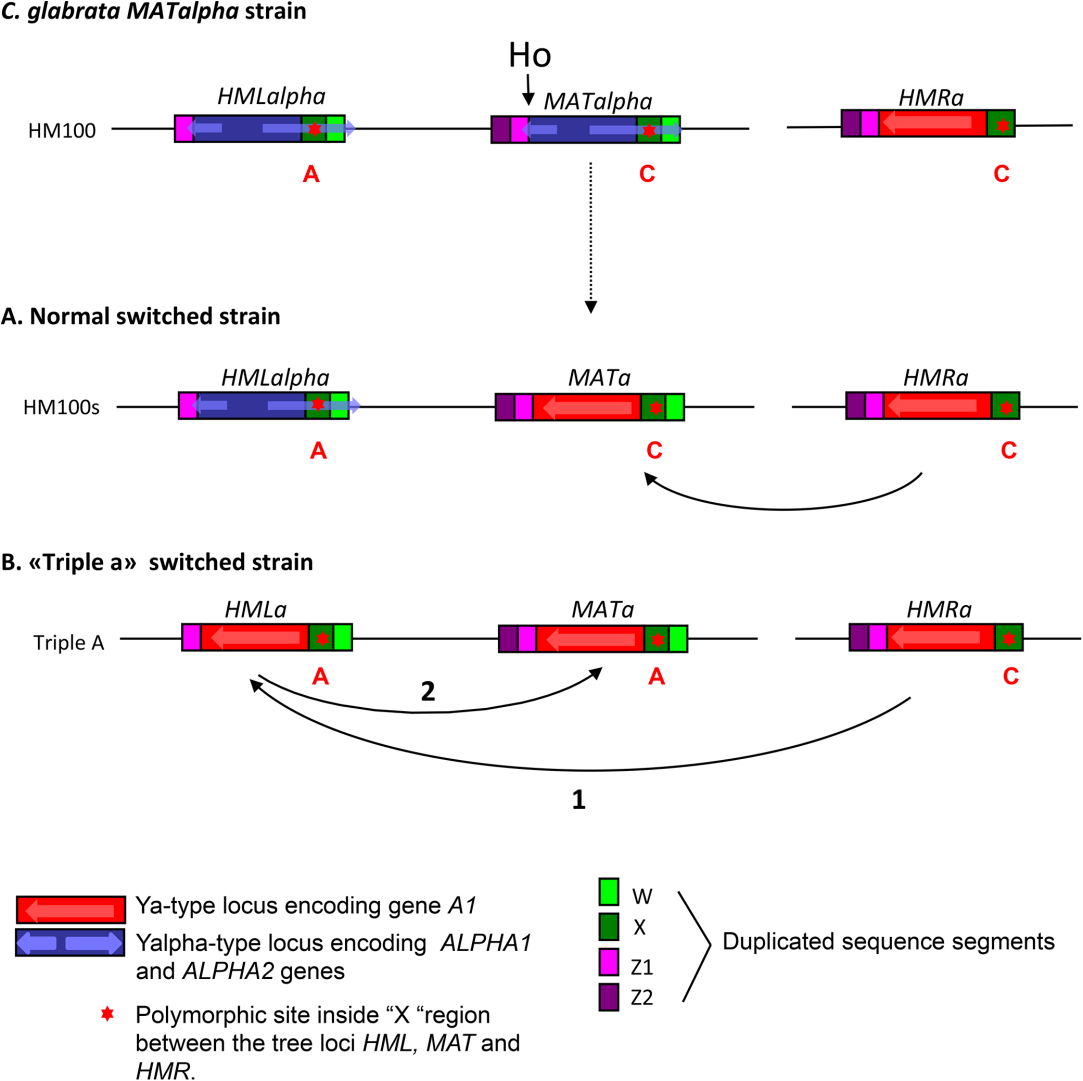
*HML* as donor and recipient. Example of a “triple a” strain in which the order of repair can be followed thanks to polymorphic sites. (See text).

Sequencing of *HML* and *HMR* loci showed that half of the clones display a normal structure of these loci (Fig 6A); but others present switching at *HML*, *i*.*e*, *HMLalpha* has been switched to *HMLa*, leading to a “triple a” genotype: *HMLa*, ***MATa***, ***HMRa*** (Fig 6B). Normal and “triple a” genotypes were confirmed by sequencing and by Southern blot experiments (data not shown). The finding of these “triple a” strains is evidence of cutting at the *HML* locus by *S*. *cerevisiae*'s Ho endonuclease.

Since the original ***MATa*** and *MATalpha* strains at our disposal were non isogenic, we decided to use a switched BG87s *MATalpha* strain to test whether “triple a” strains could be obtained in a different strain background (experiment 1-b, Table 5). We isolated two ***MATa*** BG87s clones, upon *S*. *cerevisiae’s HO* expression, confirming that the *MATalpha* switched locus can switch back to ***MATa***. Sequencing shows that the first clone displays a normal structure at the ***MATa***, *HMLalpha*, and ***HMRa*** loci, but that the second one exhibits a “triple a” structure: ***MATa***, *HMLa*, ***HMRa***, similar to the one obtained in the HM100 strain (data not shown).

### The *HML* locus can act first as a recipient and then as a donor during mating-type switching

Sequence comparison showed the presence of a polymorphic site inside the X region between the three loci, (*MAT*, *HML* and *HMR*) in the HM100 strain (Fig 6). The *MAT* and *HMR* loci possess a “C” whereas the *HML* locus presents an “A” at the same position (+1269 from the Ho cut site). In *S*. *cerevisiae*, we know that copying of *MAT* information during switching, includes not only the Y region but extends in the X region with variable lengths [67]. We took advantage of this polymorphic site to understand the chronological steps of the establishment of the “triple a” strains. Sequencing of one “triple a” strain shows that the *MAT* locus contains an “A” at the polymorphic site. This result proves that the *MAT* locus has been cut and necessarily repaired using *HML* as a template after it has switched itself, using ***HMRa*** as template. This is unexpected because it suggests that *HML* is preferentially cut over *MAT*.

### Switching-induced cell death in *C*. *glabrata*

Comparison of colony numbers between inductive and repressive conditions shows that *S*. *cerevisiae HO* gene expression induces a very strong lethality in *C*. *glabrata* cells (Table 6). This lethality is not observed during the *Nakaseomyces HO* gene expression. We observed the same rate of lethality between the two strains BG87 ***MATa*** and BG87s *MATalpha* (experiments 1 and 1-b respectively, Table 6) suggesting that the switching direction (***MATa*** *versus MATalpha*) does not influence cell death. We noticed that, in the HM100 strain background, lethality is ten times higher (Table 6, experiment 8).

**Table 6:** Estimate of cell death according to the *HO* gene expressed.

	Experiment	Strain	*MAT*	*HO* gene	Survival
*C*. *glabrata*	A	BG87	a	Without *HO* gene	100 %
	1	BG87	a	*S*. *cerevisiae*	0.1 % *
	2	BG87	a	*C*. *glabrata*	100 %
	3	BG87	a	*C*. *bracarensis*	100 %
	4	BG87	a	*C*. *nivariensis*	100 %
	5	BG87	a	*C*. *castellii*	100 %
	6	BG87	a	*N*. *bacillisporus*	100 %
	7	BG87	a	*N*. *delphensis*	100 %
	8	HM100	alpha	*S*. *cerevisiae*	0.01 %*
	1-b	BG87s	alpha	*S*. *cerevisiae*	0.1 %*
S. *cerevisiae*	21	FY1679-18D	a	*S*. *cerevisiae*	100 %
	22	FY1679-18D	a	*C*. *glabrata*	100 %

In the case of lethality (*), dilutions from 10^6^ to 10^2^ cells were plated on inductive medium and on repressive medium, and numbers of colonies compared. For experiments 1 and 1-b, number of cells counted, is between approximately 4 and 300 clones for dilutions ranging from 10^3^ to 10^5^ cells per plate. For experiment 8, we counted between 1 and 50 colonies for dilution ranging from 10^4^ up to 10^6^ cells per plate. This was done on three and five independent transformants for strains BG87 and HM100 respectively.

The high lethality observed could be due to cutting of an Ho site outside of the triplicated cassettes, which could not be repaired by homologous recombination. We unsuccessfully searched the entire genome *in silico*, for additional Ho sites, even though we cannot exclude the presence of degenerate sites. Since we observe unexpected switching of *HML* (triple “a” genotype, see above), we hypothesize that the high lethality is linked to high efficiency of cutting at the Ho sites, including the *HML* site which can be cut in *C*. *glabrata* (see discussion).

### *S*. *cerevisiae* and *C*. *glabrata HO* gene expression in *S*. *cerevisiae*

We were intrigued by the strong lethality observed in *C*. *glabrata* cells, when the *HO* gene from *S*. *cerevisiae* was expressed. Such lethality has not been reported, to our knowledge, when *HO* is expressed from a galactose-inducible promoter in a wild-type strain of *S*. *cerevisiae*. We therefore wanted to test whether our construction, with the *HO* gene under the control of the *MET3* promoter from *C*. *glabrata* could induce lethality in *S*. *cerevisiae*, taking advantage of the fact that ARS sequences can function as origins in both species [68].

A ***MATa*** *ho S*. *cerevisiae* strain (FY1679-18D) was transformed with the two constructs used previously, carrying either the *C*. *glabrata* or *S*. *cerevisiae HO* gene (experiments 21 and 22, Tables 5 and 6). Several independent transformants underwent the same treatment as *C*. *glabrata* cells, *i*.*e* they were grown in broth in repressive conditions, and plated on either inductive or repressive medium at various concentrations. We confirmed the stability of the plasmid in *S*. *cerevisiae* by comparing the number of colonies on medium with or without uracil. In *S*. *cerevisiae*, switching events were searched for, by a mating assay with a complementing strain possessing the same *MAT* type, so that mating is possible only if switching has occurred. We observed that expression of *S*. *cerevisiae*'s *HO* leads to switching of the ***MATa*** locus of *S*. *cerevisiae*, whereas no switch is observed when the *C*. *glabrata HO* gene is expressed (experiments 21 and 22, Table 5).

No lethality is observed upon expression of either *HO* genes in *S*. *cerevisiae* (experiments 21 and 22, Table 6). Thus, the strong lethality observed in *C*. *glabrata* is not due to a toxic effect of our *S*. *cerevisiae HO* construct, but seems to be specific to *C*. *glabrata* cells.

## Discussion

In *S*. *cerevisiae*, mating-type switching is a complex system relying on the existence of: 1) three cassettes (donors and recipient), 2) the presence of the Ho endonuclease 3) repair mechanisms, 4) silencing mechanisms at *HMLalpha* and ***HMRa***. These silencing mechanisms allow the switching system to work with the *MAT* locus as the sole recipient and *HMLalpha* and ***HMRa*** as donors.

*C*. *glabrata* has never been shown to mate nor to switch mating-types naturally. Its genome has retained the three cassettes and the *HO* gene, but misses the *SIR1* gene, involved in silencing of *HML* and *HMR* in *S*. *cerevisiae*. In this work, we address the functionality of different Ho proteins and of the switching system in *C*. *glabrata* by expressing all *HO* genes from the *Nakaseomyces* and *S*. *cerevisiae*. Our induction experiments are done on solid medium, so that the *HO* genes are expressed continuously. Studies in *S*. *cerevisiae* were performed in the same way (for review, [5]) and allowed the dissection of the *MAT*-switching mechanisms. This is very different from the natural situation in which *HO* gene expression is tightly regulated, and could lead to continuous cleavage during cell growth on plates. Nonetheless, in our experiments, we obtained a very reproducible proportion of switched colonies. This seems to show that the Ho proteins do not cut repeatedly and do not induce switching back and forth between ***MATa*** and *MATalpha* in our conditions.

*HO* genes from the *Nakaseomyces* and *S*. *cerevisiae* allow *MAT* switching in a ***MATa*** *C*. *glabrata* strain (BG87). We observe that the *Nakaseomyces’* Hos are poorly efficient, even those of the two mating-competent species of the clade, *N*. *delphensis* and *N*. *bacillisporus*. Unexpectedly, *S*. *cerevisiae’s* Ho is the most efficient to induce *MAT* switching in *C*. *glabrata* and we thus focused the rest of our work on this endonuclease.

*S*. *cerevisiae*’s Ho is efficient in both directions and in both genetic backgrounds (strains BG87 and HM100). Thus, the localization of *HMR* on another chromosome from *MAT* does not prevent its role as donor during double-strand break repair in *C*. *glabrata*. In addition, inducing the switch in the two backgrounds (BG87 and HM100) allows us to obtain ***MATa*** and *MATalpha* isogenic strains, which could be useful for mating assays.

In addition to its high efficiency of switch, *S*. *cerevisiae’s* Ho induces a very high cell lethality in *C*. *glabrata*, which is not the case for the *Nakaseomyces HO* genes. Moreover, this strong lethality seems to be specific to *C*. *glabrata* since 100% of *S*. *cerevisiae’s* cells survive during *S*. *cerevisiae’s HO* gene expression in the same conditions. Thus the lethality is not due to any construct toxicity or *HO* gene expression conditions. The same survival rate is observed in both sexual types BG87 (***MATa***) and BG87s (*MATalpha*), so that using the *HMR* locus as template does not seem to be involved in the observed lethality.

Switching in a *MATalpha* strain results in numerous clones which have also switched at *HML*, proof that Ho also cuts at this locus. On the contrary, we do not observe switching at *HMR* in our conditions. We cannot exclude that *HMR* is atypically cleaved by Ho, like *HML*, and that this leads to repair without switch, so that we cannot detect it. The fact that we observe cutting at *HML* and not at *HMR* is unexpected since *HMR* is transcriptionally leaky, contrary to *HML* [38]. Therefore, accessibility to transcription factors and accessibility to cleavage by Ho do not necessarily go hand in hand. In *S*. *cerevisiae*, extensive studies have revealed that *HML* and *HMR* are silenced by several proteins (mainly Sir proteins) and usually protected from Ho cleavage (for review, [5]). In *sir*- mutants, the two *HML* and *HMR* loci are unsilenced and can act as recipients and thus, switch [27–29]. Furthermore, cleavage at *HML* has been previously reported at a very low rate during constitutive *HO* expression in wild-type cells while none was observed at *HMR* [69], in accordance to what we observed in *C*. *glabrata*.

Natural variants carrying opposite configuration at the *HML* locus have been described previously in *C*. *glabrata* [36]. We screened 100 different strains of our *C*. *glabrata* collection [35] by typing the Ya or Yalpha information at the three loci *MAT*, *HMR* and *HML* by specific PCR amplifications (data not shown). We found 10% of the strains revealing rearrangements, with at least one strain harboring the *HMLa* genotype. These data confirm that cuts outside of *MAT* can occur in natural conditions.

Our results show, that in a wild-type strain of *C*. *glabrata*, both *MAT* and *HML* are cut at high levels by Ho during switching. We believe that this “illegal” cleavage is the result of a silencing system which is less efficient than *S*. *cerevisiae*’s, possibly due to the absence of *SIR1* and that this may be the cause of the mortality. *C*. *glabrata* does not follow the rule of “one recipient for two opposite donors”, essential for the proper functioning of the switching system in *S*. *cerevisiae*. The high mortality associated to efficient switching, explains perhaps, why *C*. *glabrata* is not observed to switch regularly. Our work, relying on deregulation of the genes, shows that the low efficiency of the endogenous system is probably not due to a weak endogenous promoter, but may be linked to the properties of the protein itself, such as stability, nuclear localization and/or cleavage activity. Nonetheless, the fact that the genome of *C*. *glabrata* has retained the elements necessary for switching as well as the genes involved in mating, point to the possibility that undiscovered conditions may induce switching and/or mating.

Further experiments are under way to understand the correlation between switching and cell death in *C*. *glabrata*. Especially, mutations in the Ho recognition sites should allow us to understand better the switching system and the rules for cleavage of recipients and for donor preference in this pathogen.
